# Investigation of Lysozyme Diffusion in Agarose Hydrogels Employing a Microfluidics-Based UV Imaging Approach

**DOI:** 10.3389/fbioe.2022.849271

**Published:** 2022-03-08

**Authors:** Lukas Wenger, Jürgen Hubbuch

**Affiliations:** Institute of Process Engineering in Life Sciences, Section IV: Biomolecular Separation Engineering, Karlsruhe Institute of Technology, Karlsruhe, Germany

**Keywords:** diffusion, agarose, hydrogels, Fick’s law, microfluidics, UV area imaging, image processing, ActiPix

## Abstract

Hydrogels are polymer-based materials with a high water content. Due to their biocompatible and cell-friendly nature, they play a major role in a variety of biotechnological applications. For many of these applications, diffusibility is an essential property influencing the choice of material. We present an approach to estimate diffusion coefficients in hydrogels based on absorbance measurements of a UV area imaging system. A microfluidic chip with a y-junction was employed to generate a fluid-hydrogel interface and the diffusion of lysozyme from the fluid into the hydrogel phase was monitored. Employing automated image and data processing, analyte concentration profiles were generated from the absorbance measurements and fits with an analytical solution of Fick’s second law of diffusion were applied to estimate diffusion coefficients. As a case study, the diffusion of lysozyme in hydrogels made from different concentrations (0.5–1.5% (w/w)) of an unmodified and a low-melt agarose was investigated. The estimated diffusion coefficients for lysozyme were between 0.80 ± 0.04×10^−10^ m^2^ s^−1^ for 1.5% (w/w) low-melt agarose and 1.14 ± 0.02×10^−10^ m^2^ s^−1^ for 0.5% (w/w) unmodified agarose. The method proved sensitive enough to resolve significant differences between the diffusion coefficients in different concentrations and types of agarose. The microfluidic approach offers low consumption of analyte and hydrogel and requires only relatively simple instrumentation.

## 1 Introduction

Hydrogels are polymer-based materials with a high water content (Ahmed, 2015). They are employed in a variety of medical and biotechnological applications like the immobilization of enzymes (Russell et al., 1999; Kunkel and Asuri, 2014), tissue engineering (Zhao et al., 2016; Spicer, 2020) or as bioinks in bioprinting (You et al., 2017; Rastogi and Kandasubramanian, 2019). Both synthetic polymers like poly(ethylene glycol)-diacrylate (Tan et al., 2012) or poly(vinyl alcohol) (Gibas et al., 2010; Zhang et al., 2012) and natural polymers like agarose (Rahfoth et al., 1998), alginate (Tan and Takeuchi, 2007) or gelatin (Sheelu et al., 2008) can serve as the base material of hydrogels. The aqueous matrices of hydrogels mimic native soft tissues and provide a cell-friendly and highly biocompatible environment (Spicer, 2020) that is ideally suited to accommodate living cells or stress-sensitive biomolecules like enzymes. An essential property of hydrogels that is relevant for many applications is their diffusibility. In tissue engineering, cells embedded in or growing on top of hydrogels rely on the diffusional transport of oxygen, nutrients and waste products through the hydrogel to sustain their metabolism (Lovett et al., 2009). In biocatalytic applications involving physically entrapped enzymes, the hydrogel should ensure the retention and immobilization of the relatively large enzyme within the hydrogel, while allowing the diffusion of small substrate and product molecules (Krishnamoorthi et al., 2015). The diffusion of a molecule through a hydrogel depends mainly on the size of the molecule, the crosslinking density of the hydrogel polymer network (Weber et al., 2009) and potential physical interactions between the diffusing molecule and the polymer chains of the hydrogel. This includes van der Waals forces (Weber et al., 2009) and electrostatic interactions (Hirota et al., 2000; Ye et al., 2016).

Precise knowledge about the diffusional behavior of compounds in hydrogels is essential for many applications. A large variety of methods for the determination of diffusion coefficients has been described. Methods like fluorescence recovery after photobleaching (FRAP) (Pluen et al., 1999; Deschout et al., 2010; Hagel et al., 2013), dual-focus fluorescence correlation spectroscopy (FCS) (Müller et al., 2008) or the tracking of diffusing molecules using a fluorescence microscope (Hettiaratchi et al., 2018) are only applicable to fluorescent compounds or require fluorescent tagging of the target molecule which may alter the compound’s behavior and requires additional conjugation and purification steps (Teske et al., 2005). Holographic laser interferometry (HLI) (Gustafsson et al., 1993; Mattisson et al., 2000), electron speckle pattern interferometry (ESPI) (Karlsson et al., 2002) and pulsed-field-gradient nuclear magnetic resonance (PFG-NMR) (Gibbs et al., 1992; Harmon et al., 2012) require specialized and costly equipment not typically available in most laboratories. Other methods like Taylor dispersion analysis (TDA) (Ye et al., 2012a; Jensen et al., 2014), refractive index methods (Liang et al., 2006) or certain spectrophotometric methods (Dunmire et al., 1999; Di Cagno et al., 2018) may suffer from limitations like requiring relatively large sample volumes or being only applicable for liquid samples.

UV area imaging systems have been employed for the spatial observation of diffusion processes in hydrogels to estimate diffusion coefficients. Studies with several different compounds and hydrogels have been conducted, e. g. addressing the diffusion of human serum albumin and piroxicam in Pluronic F127 hydrogels (Ye et al., 2011), piroxicam in subcutaneous tissue models based on agarose and Pluronic F127 (Ye et al., 2012b), insulin in agarose hydrogels (Jensen et al., 2014) and lysozyme and several other proteins in different agarose-based cartilage models (Ye et al., 2016). The described methods consume relatively large amounts of sample due to the use of quartz cells requiring hydrogel sample volumes between 310 and 600 µl. The high amount of material consumption may prevent these methods from being applied with cost-intensive materials or materials with limited availability, as scaling effects caused by high numbers of experiments and replicates may render the methods prohibitively expensive. Indeed, commercially available bioinks may cost more than a hundred dollars for 1 ml of hydrogel. Even more challenging may be the often very limited availability of novel, custom-made materials only synthesized on a small lab scale.

**FIGURE 1:**
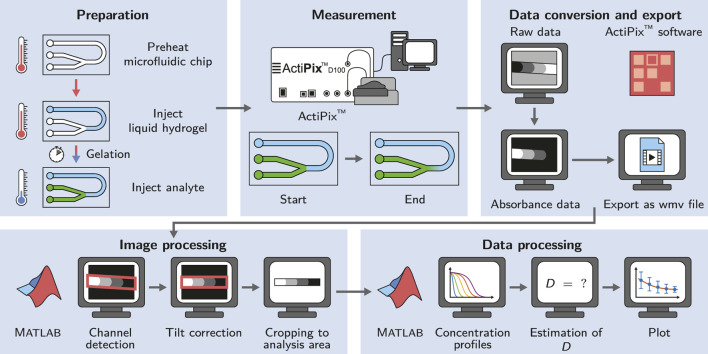
Schematic of the workflow applied in this study. The microfluidic chip is preheated to allow the injection of liquid agarose into the channel and ensure the controlled gelation by cooling at room temperature. The diffusion measurement is started right after the injection of the analyte. The acquired data is converted and exported using the ActiPix™ software. Image and data processing for the estimation of diffusion coefficients is done in Matlab.

We present a modified approach employing a microfluidic chip to observe the diffusion of an analyte within a hydrogel-filled microchannel using an ActiPix™ UV area imaging system. The presented technique reduces both hydrogel and analyte consumption down to 40 and 25 μl, respectively. Automated image processing and data evaluation is employed to analyze the acquired raw absorbance data provided by the imaging system. Diffusion coefficients are estimated by fitting a solution of Fick’s second law with obtained analyte concentration profiles along the channel. The applicability of the method is demonstrated by a case study involving lysozyme as the analyte and hydrogels made from different concentrations (0.5–1.5% (w/w)) of an unmodified and a low-melt agarose. The applied workflow of the study is schematically summarized in Figure 1.

## 2 Materials and Methods

### 2.1 Manufacturing of the Microfluidic Chip

**FIGURE 2:**
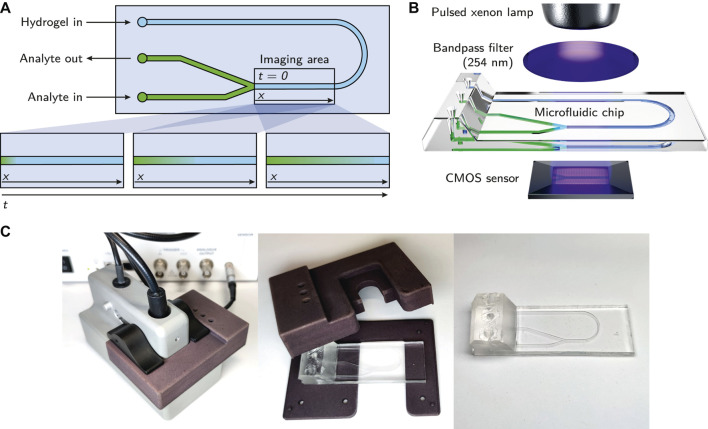
**(A)** Schematic of the microfluidic chip. The imaging area being observed by the ActiPix™ is roughly indicated with a rectangle. The bottom part of the subfigure shows the observed diffusion of the analyte through the hydrogel over time. **(B)** Overview of the essential components of the employed setup. The illustration demonstrates the integration of the microfluidic chip within the Actipix™ imaging system. Absorbance area measurements are generated by guiding light from a pulsed xenon lamp through a 254 nm bandpass filter and the microfluidic chip before being detected by a CMOS sensor. **(C)** Photographs of the microfluidic chip with the casing.

A simple microfluidic chip with three inlets and a y-junction was custom-made using a silicone molding technique as described by Waldbaur *et al.* (Waldbaur et al., 2013). In short, a specific replication master made by stereolithography was kept in place by a molding tool and a spacer to allow the casting of the top part of the microfluidic chip. To achieve a smooth and clear surface suitable for absorbance measurements, a spacer made from polished stainless steel was used (Radtke et al., 2016). Elastosil^®^RT 601 (Wacker Chemie AG), a pourable, two-component silicone rubber that cures at room temperature was used as the base material for the chip. After curing, the silicone part was bonded with a second, planar silicone part to seal the channels. To achieve bonding, the silicone surface was activated by plasma treatment with a hand-held corona treater (BD-20AC, Electro-Technic Products Inc.) (Haubert et al., 2006). The channels were 1,000 µm in width and 500 µm in height. A scheme of the employed microfluidic chip is shown in Figure 2A, a 3D rendering in Figure 2B and photographs in Figure 2C.

### 2.2 Chemicals and Buffer Preparation

Unmodified agarose (Roti^®^garose for DNA/RNA electrophoresis) and low-melt agarose (Roti^®^garose with low melting and gelling temperature) were obtained from Carl Roth GmbH & Co. KG. Lysozyme (lyophilized, from chicken egg white, Hampton Research) was used for the analyte solution. The hydrogels and lysozyme solutions were prepared with phosphate buffered saline (PBS), pH 7.4. Sodium chloride (NaCl), potassium chloride (KCl), disodium hydrogen phosphate dihydrate (Na_2_HPO_4_ ⋅ 2H_2_O) and potassium dihydrogen phosphate (KH_2_PO_4_) were purchased from Merck KGaA. The PBS buffers were prepared with ultrapure water from a Purelab Ultra water purification system (ELGA LabWater) and filtered through an 0.2 µm cellulose acetate filter (Sartorius AG).

### 2.3 Hydrogel Preparation

Agarose hydrogels were prepared at different concentrations (0.5, 1.0 and 1.5% (w/w)) in PBS buffer, pH 7.4. Appropriate amounts of agarose powder were dissolved in PBS buffer by heating up the mixtures several times to boiling point using a microwave oven (WP800L20-5, Hanseatic). The resulting solutions were transferred to preheated syringes, sealed and stored at 60°C until use in a drying oven (T6120, Heraeus Instruments) to avoid gelation.

### 2.4 Preparation of the Microfluidic Chip for the Measurement

The microfluidic chip was preheated at 60°C in a drying oven (T6120, Heraeus Instruments) for 10 min. Liquid agarose solution (∼40 µl) was injected into the preheated chip using a blunt needle (Sterican^®^ MIX, 1.2 × 40 mm, B. Braun SE), until it reached the Y-junction of the channel, as shown in Figure 2A. The chip was left at room temperature for 10 min to cool down and allow the gelation of the hydrogel. This duration was chosen based on experiments with 0.5% (w/w) low-melt agarose where a gelling time of 5 min was found to be sufficient to avoid dissolving the gel when injecting buffer into the other inlets of the chip. After gelation, the analyte solution (∼25 µl) was injected into the chip through one of the other inlets immediately before the start of the measurement, generating an interface between analyte solution and hydrogel at the y-junction. The y-junction design was chosen to allow the air contained in the microfluidic channel to escape during the injection of the analyte solution.

### 2.5 Diffusion Measurements: UV Imaging and Data Export

An ActiPix™ D100 UV area imaging system (Paraytec Ltd.) was employed to observe the propagation of the analyte through the hydrogel over time. It was equipped with a pulsed xenon lamp, a 254 nm bandpass filter and a complementary metal-oxide-semiconductor (CMOS) sensor for detection. A simplified schematic of the parts of the setup is shown in Figure 2B. The microfluidic chip was positioned in the appropriate location using a custom-made, 3D-printed casing which provided access to the channel inlets and shielded the chip from external light sources. To reduce the potential influence of stray light further, all measurements were performed in a darkened room and the inlets of the chip were sealed with aluminium foil. With the employed setup, the imaging area (9 × 7 mm, 1,280 × 1,024 pixels) was limited to the region of the microfluidic chip immediately after the y-junction, as indicated in Figure 2A.

Each measurement was started immediately after the injection of the analyte solution. After dark images and reference images were collected for 60 s each, imaging data were collected for 4 h at a frame rate of 0.18 s^−1^. All measurements were performed at an ambient temperature of 22 ± 2°C. Using the ActiPix™ software version 1.5 (Paraytec Ltd.), the acquired data were converted to absorbance data and exported as wmv (Windows Media Video) files for further processing.

### 2.6 Image Processing

**FIGURE 3:**
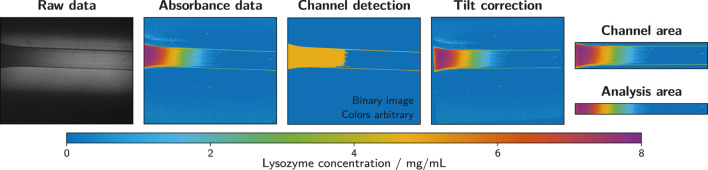
Image processing steps as performed with the ActiPix™ software and Matlab. The example shows the last frame of a measurement with 10 mg/ml lysozyme as the analyte solution and 1.5% (w/w) low-melt agarose as the hydrogel. Raw data files were converted to absorbance data using the ActiPix™ software. In Matlab, the channel was detected based on the last frame of the measurement to correct for inaccuracies in positioning and alignment. Based on the channel detection, the tilt was corrected and the channel area extracted. An area of 100 pixels in height was taken from the middle of the channel and used as the analysis area.

The exported wmv files containing the collected absorbance data were processed in Matlab R2020a (The MathWorks^®^, Inc.) using an automated script. The raw video files were read in and converted to grayscale. Due to the flexibility of the microfluidic silicone chip and some clearance between the chip and the casing, the orientation of the channel deviated from a perfect horizontal alignment in most measurements. This deviation was constant during the measurement, hence the last frame of each measurement was evaluated to provide a suitable correction. The channel was detected using a thresholding function and its orientation was determined. Each frame of the measurement was rotated by the appropriate angle to obtain a horizontally aligned channel. To reduce the image size to the relevant minimum, each frame was cropped to an area of 100 pixels in height containing the middle of the channel over the whole observed channel length. This extracted part of the frame served as the analysis area that provided the data for the generation of analyte concentration profiles along the channel over time (see Section 2.8.1). The described image processing steps are exemplarily illustrated in Figure 3.

### 2.7 Calibration Curves

Calibration curves with different lysozyme concentrations (0 mg/ml to 10 mg/ml) were prepared by filling the channel of the microfluidic chip with the respective analyte solution and recording the absorbance for 5 min. Measurement, data export and image processing steps were performed in the same way as for the diffusion measurements (Section 2.5 and 2.6). To obtain a value for the calibration curve, a mean pixel intensity value was calculated from a part of the analysis area (100 × 100 pixels) of the last frame of each measurement. PBS buffer (0 mg/ml lysozyme) served as a blank. All data points of the calibration were in the linear range of Beer’s law.

### 2.8 Data Processing

#### 2.8.1 Determination of Concentration Profiles

**FIGURE 4:**
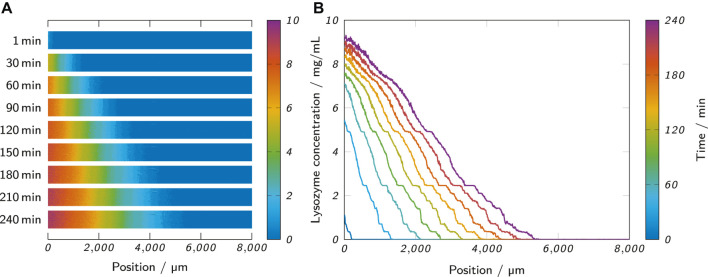
**(A)** The observed analysis area of a measurement at different time points shows the propagation of the lysozyme through the hydrogel. **(B)** The lysozyme concentration profiles of the same measurement were obtained from the analysis area by column-wise calculation of mean values. The shown measurement was performed with 0.5% (w/w) low-melt agarose as the hydrogel and 10 mg/ml lysozyme as the analyte solution.

The extracted and corrected image data of the analysis area were further processed in Matlab R2020a. A blank value was obtained from the end part of the analysis area of the first valid data frame. This area (100 × 100 pixels) covered a part of the channel that only contained hydrogel, but no analyte (*c*_*lysozyme*_ = 0). The mean of the pixel intensities of this region served as a blank value and was subtracted from the image data of all frames. From each 240 min long measurement, a frame was extracted every 15 min, starting at 30 min. All absorbance values were converted to lysozyme concentration values using the obtained calibration curves. Figure 4A exemplarily shows the extracted and converted analysis areas of a measurement with 0.5% low-melt agarose at different time points. To create lysozyme concentration profiles along the channel length, column-wise mean values were calculated with one column corresponding to 7 µm of channel length. Figure 4B shows the resulting lysozyme concentration profiles of the extracted frames of the same measurement.

#### 2.8.2 Estimation of Diffusion Coefficients Using Fick’s Second Law

To estimate diffusion coefficients from the recorded concentration profiles, diffusion in one dimension (along the microfluidic channel) according to Fick’s second law of diffusion was assumed (Crank, 1975):

$$\frac{\partial C}{\partial t}=D\frac{\partial^{2}C}{\partial x^{2}}$$ (1)

where *C* is the analyte concentration, *t* is the time, *x* is the distance along the channel and *D* is the diffusion coefficient of the analyte in the medium. Different analytical solutions for Fick’s second law exist, depending on the assumed boundary and initial conditions (Crank, 1975). To find a solution suitable to describe the observed concentration profiles, three different solutions were applied in this study, all assuming a system of two phases (in the present case fluid and hydrogel phase) with different analyte concentrations. The diffusion coefficient *D* is assumed to stay constant during the measurement. The initial (*t* = 0) concentration of analyte is *C*_0_ in the fluid phase (*x* < 0) and zero in the hydrogel phase (*x* > 0). The phases are in contact at a boundary layer at *x* = 0. The solutions assume either both the fluid-containing and the hydrogel-containing channel as infinitely long (infinite system) or only the hydrogel-containing channel (semi-infinite system) with a constant analyte concentration *C*_0_ at the boundary layer. The assumption of infinity demands the condition *t* ≪ *L* ^2^/*D* to be met, with the duration of the measurement *t* and the length of the hydrogel-filled channel *L*. This assumption implies that the analyte concentration at the end of the measurement is still zero at the end of the hydrogel-filled channel and still *C*_*O*_ at the end of the fluid-filled channel (Ye et al., 2012b).

The first applied solution assumes a composite system composed of two phases with different diffusion coefficients. Assuming no accumulation of the analyte at the boundary layer (*x* = 0), the boundary condition can be expressed as follows (Crank, 1975; Ye et al., 2012b):

$$D_{hydrogel}\frac{\partial C_{hydrogel}}{\partial x}=D_{\mathrm{fl}uid}\frac{\partial C_{\mathrm{fl}uid}}{\partial x}$$ (2)

with the diffusion coefficients and concentrations of the analyte in the hydrogel (*D*_*hydrogel*_ and *C*_*hydrogel*_) and in the fluid, respectively (*D*_*fluid*_ and *C*_*fluid*_). From this, a solution for the analyte concentration in the hydrogel phase (*x* > 0) can be derived (Ye et al., 2012b):

$$C\left(x,t\right)=\frac{C_{0}\sqrt{\frac{D_{\mathrm{fl}uid}}{D_{hydrogel}}}}{1+K\sqrt{\frac{D_{\mathrm{fl}uid}}{D_{hydrogel}}}}\left(1-erf\left(\frac{x}{2\sqrt{D_{hydrogel}t}}\right)\right)$$ (3)

with the equilibrium ratio of analyte concentration between the two phases *K* = *C*_*fluid*_/*C*_*hydrogel*_.

The other two applied solutions do not assume a composite system with two different values for *D*, but a constant *D* within the whole system. The second solution assumes a semi-infinite medium with a boundary layer that is kept at a constant analyte concentration *C*_0_ throughout the whole measurement (*t* > 0). The analyte concentration in the hydrogel-filled channel over time *t* and distance *x* is then given by (Crank, 1975):

$$C\left(x,t\right)=C_{0}\left(1-erf\left(\frac{x}{2\sqrt{Dt}}\right)\right)$$ (4)

The third applied solution of Fick’s second law assumes an infinite system with the fluid phase (*x* < 0) acting as an extended analyte source of infinite extent. With the initial conditions *C* = *C*_0_ in the fluid phase and *C* = 0 in the hydrogel phase (*x* > 0), the analyte concentration is given by (Crank, 1975):

$$C\left(x,t\right)=C_{0}\left(\frac{1}{2}-\frac{1}{2}erf\left(\frac{x}{2\sqrt{Dt}}\right)\right)$$ (5)

In this case, the concentration at the boundary layer is $\frac{1}{2}C_{0}$ throughout the measurement and diffusion processes take place on both sides of the boundary layer.

As mentioned before, all equations assume a fixed boundary layer at the position *x* = 0. In practice, the employed experimental setup did not allow a precise and reproducible positioning of the hydrogel-fluid interface at *x* = 0 for each measurement. Differences in positioning of the microfluidic chip and slightly varying hydrogel fill levels introduced a variability that was accounted for by introducing an additional parameter *x*_0_. This allowed expressing Eqs 3, 4, and 5 with a variable position of the boundary layer at *x* = *x*_0_ (Ye et al., 2011):

$$\begin{array}{ll} in\mathrm{fi}nitecompositesystem: & C\left(x,t\right)=\frac{C_{0}\sqrt{\frac{D_{\mathrm{fl}uid}}{D_{hydrogel}}}}{1+K\sqrt{\frac{D_{\mathrm{fl}uid}}{D_{hydrogel}}}}\left(1-erf\left(\frac{x-x_{0}}{2\sqrt{D_{hydrogel}t}}\right)\right) \end{array}$$ (6)

$$\begin{array}{ll} semi-in\mathrm{fi}nitesystem:C\left(x,t\right) & =C_{0}\left(1-erf\left(\frac{x-x_{0}}{2\sqrt{Dt}}\right)\right) \end{array}$$ (7)

$$\begin{array}{ll} in\mathrm{fi}nitesystem:C\left(x,t\right) & =C_{0}\left(\frac{1}{2}-\frac{1}{2}erf\left(\frac{x-x_{0}}{2\sqrt{Dt}}\right)\right) \end{array}$$ (8)

To estimate diffusion coefficients, the observed concentration profiles of all samples were fitted with the presented solutions of Fick’s second law using Matlab. For Eq. 6, a diffusion coefficient for lysozyme in the fluid phase *D*_*fluid*_ = 1.2 × 10^−10^ m^2^/s (lysozyme in 67 mM phosphate, pH 7.4 at 25°C) was assumed (Ye et al., 2016). In the course of the manuscript, Eq. 8 was finally chosen for further analysis. For a clearer overview, the assumptions and boundary conditions of the three equations are summarized in Table 1.

**TABLE 1:** Summary of the general assumptions and the boundary and initial conditions of the applied equations.

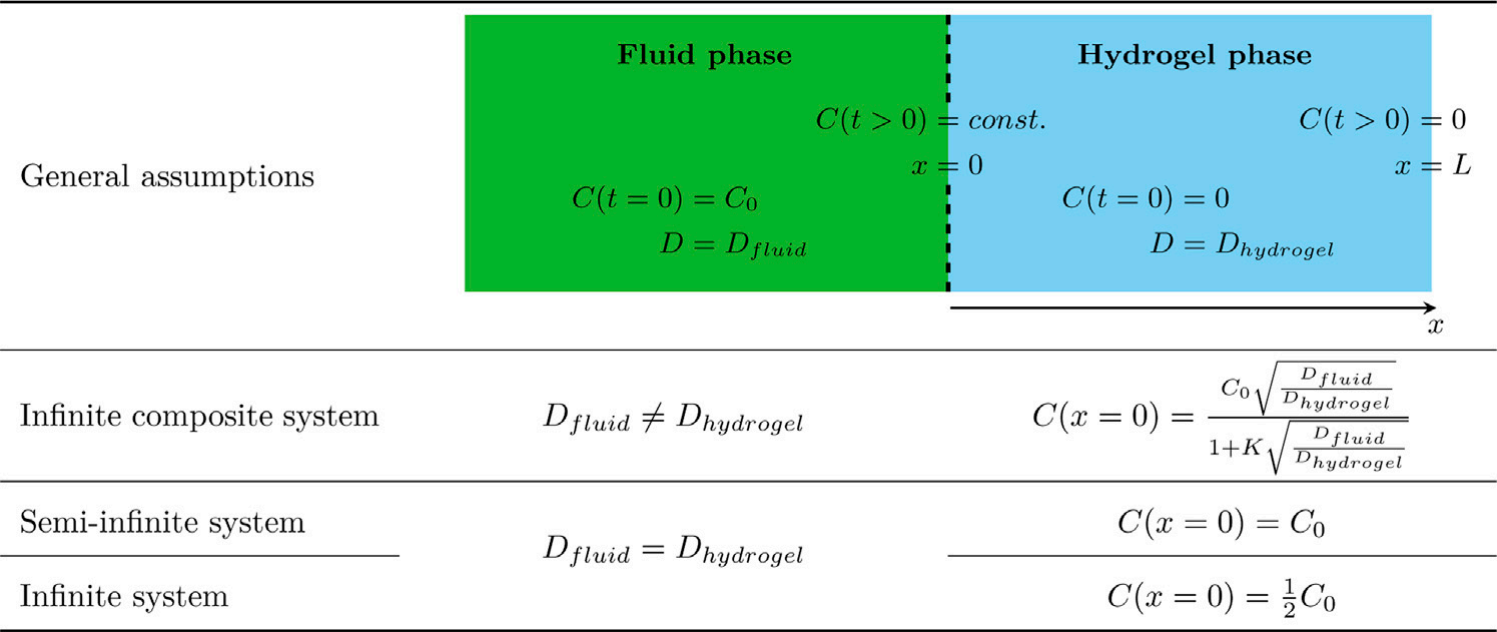

### 2.9 Influence of Temperature

All measurements were performed at an ambient temperature of 22 ± 2°C. The actual temperature within the microfluidic chip could not be determined, but it can be estimated that the employed setup involving the ActiPix™ UV imaging system generates a temperature gradient of up to 5°C compared to ambient temperature (Jensen et al., 2014). Due to the high uncertainty with regards to the actual experimental temperature (ambient temperature ±2°C, unknown temperature gradient), no corrections of the estimated *D* values were implemented, but an error estimation was performed.

The influence of temperature on the diffusion coefficient can be estimated using the Stokes-Einstein equation:

$$D=\frac{k_{B}\cdot T}{6\pi \cdot \eta \cdot r}$$ (9)

with Boltzmann’s constant *k*_*B*_, the absolute temperature *T*, the viscosity of the surrounding solution *η* and the hydrodynamic radius of the analyte *r*. Assuming a temperature difference Δ*T* = 5K with the two temperatures *T*_1_ = 22°C = 295K and *T*_2_ = 27°C = 300 K and a constant *r*, the ratio of the corresponding diffusion coefficients *D*_1_ and *D*_2_ is given by:

$$\frac{D_{1}}{D_{2}}=\frac{T_{1}\eta_{2}}{T_{2}\eta_{1}}$$ (10)

with the viscosities of the solution *η*_1_ at *T*_1_ and *η*_2_ at *T*_2_. The viscosities of the solution were estimated using an empirical model for water (Reid et al., 1987):

$$\eta =\exp\left(A+\frac{B}{T}+CT+DT^{2}\right)mPa\cdot s$$ (11)

with the constants *A* = − 24.71, *B* = 4209*K*, *C* = 0.04527 K^−1^ and *D* = − 3.376 × 10^−5^ K^−2^. Combining Eq. 10 with Eq. 11 yields:

$$\frac{D_{1}}{D_{2}}=0.88$$ (12)

The result implies an error of 12% for *D* assuming Δ*T* = 5K.

### 2.10 Statistical Analysis

The statistical significance of data was tested employing one-way analysis of variance (ANOVA) and the Tukey method for multiple comparisons. Differences between data points were considered statistically significant when *p* < 0.05.

## 3 Results and Discussion

An ActiPix™ UV imaging system was used to observe the diffusion of lysozyme through agarose hydrogels contained in the channel of a microfluidic chip. The acquired absorbance data were exported as video files and processed in Matlab to generate lysozyme concentration profiles along the length of the microfluidic channel. The diffusion coefficient of lysozyme within the agarose hydrogels was estimated by fitting the concentration profiles with three analytical solutions of Fick’s second law.

### 3.1 Choice of an Appropriate Equation for the Estimation of Diffusion Coefficients

The analytical solutions for Fick’s second law presented in Section 2.8.2 are based on certain assumptions like a constant diffusion coefficient *D* and boundary and initial conditions. These assumptions as simplifications of reality should represent the experimental setting as accurately as possible, but it is not always obvious which assumptions match a given case best. For the presented experimental setup, the boundary layer is given by the interface between fluid phase and hydrogel phase. The analyte is dissolved in the fluid phase and starts to diffuse into the hydrogel phase at the beginning of the experiment. Assuming a large and well-mixed reservoir of fluid phase or a large reservoir in combination with a diffusion coefficient that is considerably higher in the fluid phase than in the hydrogel phase (*D*_*fluid*_ ≫ *D*_*hydrogel*_), the assumption of a constant analyte concentration at the boundary as in Eq. 7 seems valid. Assuming *D*_*fluid*_ ≈ *D*_*hydrogel*_ and a stagnant fluid phase, Eq. 8 seems more appropriate, as this equation assumes diffusion processes on both sides of the boundary layer and requires a constant *D* throughout both phases. In reality, both scenarios will not ideally match the presented experimental setup, because *D*_*fluid*_ is likely to be higher than *D*_*hydrogel*_, although this difference has been reported to be only marginal or non-existent for the diffusion of insulin (Jensen et al., 2014) and lysozyme (Ye et al., 2016) in low-concentration agarose gels. Eq. 6 assumes a composite system of two materials with different diffusion coefficients and should theoretically resemble the given case best.

**FIGURE 5:**
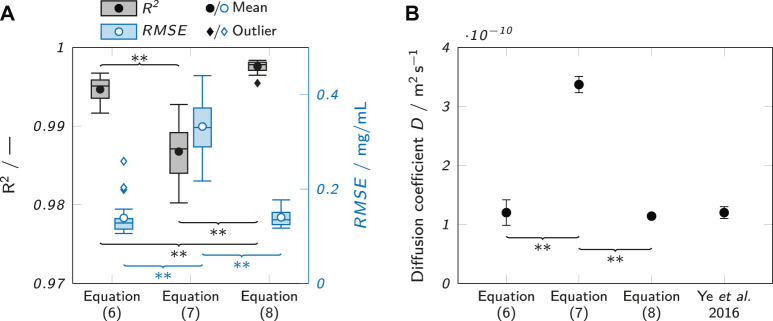
Evaluation of the suitability of different fits to describe the observed concentration profiles. **(A)** Observed *R* ^2^ values when fitting observed concentration profiles after 240 min with different analytical solutions of Fick’s second law (Eqs 6, 7 and 8). The box plots represent the median and the upper and lower quartile. The whiskers represent the most extreme value still within a 1.5-fold interquartile range (IQR) from the upper and lower quartile. All data points outside the 1.5-fold IQR are depicted as outliers. Each box represents 18 samples (*n* = 18). **(B)** Comparison of the obtained *D* estimates for lysozyme in 0.5% (w/w) agarose with each other and a value from literature (Ye et al., 2016) for lysozyme in 0.5% (w/v) agarose at 21–24°C. Significant differences between the equations are highlighted by asterisks (*p < 0.05, **p < 0.005).

In order to empirically evaluate the suitability of the equations to fit the observed data, the concentration profiles of all recorded samples were fitted with all three equations at an analysis time of 240 min. The coefficient of determination *R* ^2^ and the root-mean-square error *RMSE* of all fits were determined, as depicted in Figure 5A. The fits with Eq. 7 (semi-infinite system) only showed a mean value of 0.987 ± 0.03 for *R* ^2^, while *R* ^2^ was significantly higher at 0.995 ± 0.02 for Eq. 6 (composite system) and even higher at 0.998 ± 0.01 for fits with Eq. 8 (infinite system). The *RMSE* was 0.332 ± 0.060 mg/ml for Eq. 7. The *RMSE* values for fits with Eqs 6, 8 were about 60% lower and not significantly different from each other with 0.139 ± 0.039 mg/ml and 0.140 ± 0.018 mg/ml, respectively. However, the *RMSE* values for fits with Eq. 6 showed more outliers. This could be due to the fitting of the additional parameter *K* which may increase the likelihood of finding local instead of global fit optima.

Besides the indicators for fit quality, the suitability of the equations to describe the observed data was judged by comparing the absolute values of the determined diffusion coefficients to a literature value obtained by Ye *et al.* (Ye et al., 2016). Figure 5B shows the values obtained with the different equations in comparison to the literature value. It is clear that Eq. 7 agrees least with the reported literature value and is higher by a factor of about 3. The other two equations align well with the value from Ye *et al.*

The results identify Eq. 7 as the least suitable to describe the observed concentration profiles (low *R* ^2^, high *RMSE*) and to estimate diffusion coefficients (poor agreement with literature value). The assumption of a constant analyte concentration *C*_0_ does obviously not match the experimental conditions. Perfusing the analyte channel with a constant flow of analyte solution could change this, but would require a massively increased complexity of the experimental setup and cause a higher analyte consumption.

Equations 6, 8 performed very similarly. The obtained diffusion coefficient values were in very good agreement with data reported by Ye *et al.* (Ye et al., 2016) for both equations, but Eq. 8 generated slightly higher *R* ^2^ values and less outliers in the *RMSE* analysis indicating more robust fits. Consequently, Eq. 8 was chosen for further analysis.

**FIGURE 6:**
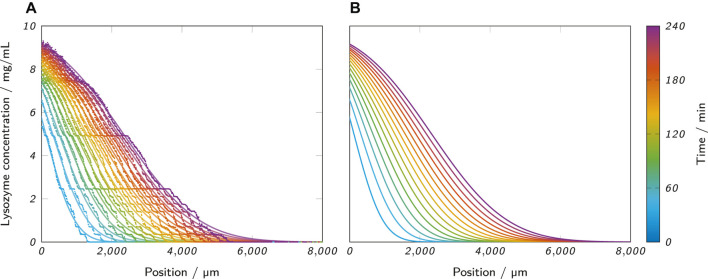
**(A)** Lysozyme concentration profiles along the microfluidic channel at the analyzed time points, as obtained from an ActiPix™ measurement. The scattered raw data are underlaid with the corresponding fits with Eq. 8. The example shows a measurement with 0.5% low-melt agarose as the hydrogel and 10 mg/ml lysozyme as the analyte solution. **(B)** Separate visualization of the same fits for better visibility.

Figure 6A shows the raw data of the recorded concentration profiles of an exemplary sample (0.5% low-melt agarose as hydrogel, 10 mg/ml lysozyme as analyte solution) at all analyzed time points. The raw data are underlaid with the corresponding fits with Eq. 8 which are also separately presented in Figure 6B. The graph visualizes the progression of lysozyme through the hydrogel and allows to draw conclusions about the analyte concentration at different penetration depths and time points. However, the observed concentration profiles clearly contradict an assumption of Eq. 8 which requires a stationary boundary layer at *x* = *x*_0_ with a constant analyte concentration of $\frac{1}{2}C_{0}$ which corresponds to 5 mg/ml lysozyme in this case. In the observed data, the concentration of 5 mg/ml moves along the *x*-axis over time implying a moving boundary. Post-measurement observations could rule out the possibility that the hydrogel-fluid interface was actually moving along the channel during the measurement due to shrinkage or other effects. Hence, the observed data suggest that Eq. 8 does not ideally represent the experimental setup. One cause for this could be the difference of the lysozyme diffusion coefficients in the fluid and the hydrogel phase. However, the same effect of a moving boundary was observed for fits with Eq. 6 which assumes a composite system with two different diffusion coefficients. Other potential reasons for the moving boundary could be undesired side-effects like capillary action, protein adsorption (Crank, 1975) or a change of the diffusion coefficient over time due to a temperature increase caused by heat dissipation from the ActiPix™ device, as reported before (Jensen et al., 2014).

### 3.2 Case Study and Influence of Analysis Time on Diffusion Coefficient Estimation

**FIGURE 7:**
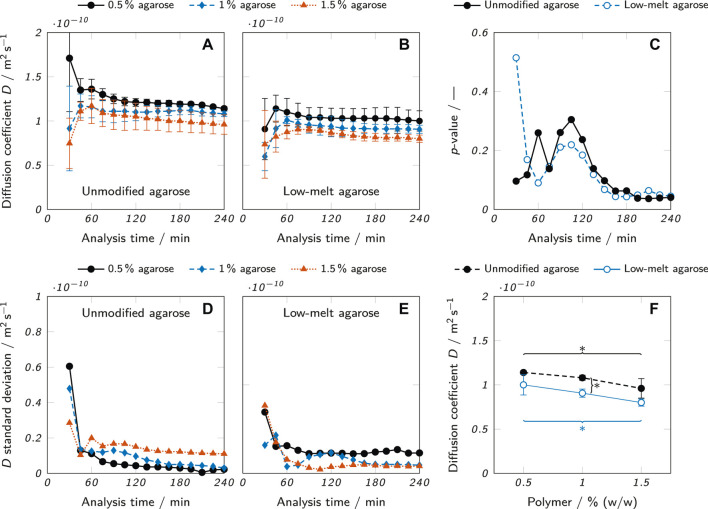
Diffusion coefficient estimates at different analysis times for **(A)** unmodified agarose and **(B)** low-melt agarose hydrogels. **(C)** *p*-values of a one-way ANOVA testing the null hypothesis that there is no difference among the mean values of *D* for samples of different agarose concentrations. (D and E) Standard deviations for **(D)** unmodified and **(E)** low-melt agarose hydrogels resulting from the *D* estimates which were carried out as triplicates (*n* = 3). **(F)** Diffusion coefficients for different concentrations of unmodified and low-melt agarose hydrogels obtained at an analysis time of 240 min. The results are presented as mean values ± standard deviation (*n* = 3). Significant differences between the Equations are highlighted by asterisks (*p < 0.05, **p < 0.005).

As a case study for the application of the presented method, the diffusion of lysozyme in agarose hydrogel samples was analyzed. Two different kinds of agarose, an unmodified agarose and a low-melt hydroxyethyl agarose at three different concentrations (0.5, 1 and 1.5% (w/w)) were examined in triplicates for 4 h per measurement. The diffusion coefficient of lysozyme in the hydrogel was estimated for each sample by fitting the measured concentration profiles with Eq. 8. To evaluate whether the apparent moving boundary had negative effects on the estimation of *D* like strongly deviating values depending on the analysis time, an investigation of the influence of analysis time was carried out. Specifically, concentration profiles were fitted and the diffusion coefficient estimated at different time points of the measurement. Figure 7A shows the results for the unmodified agarose, the corresponding standard deviations of the triplicates are plotted in Figure 7D. At an analysis time of 30 min, the standard deviations were relatively high (between 2.9 and 6.1 × 10^−11^ m^2^ s^−1^) compared to the remaining time points, indicating a lack of significant data points to perform reproducible and robust fits for several replicates. Between 45 and 240 min, the standard deviations were considerably lower ( < 2 × 10^−11^ m^2^ s^−1^) with a slight downward trend over the whole time period. As can be derived from Figure 6, the fits became more precise and more reproducible with increasing analysis time due to the increased coverage of data points over the whole course of the sigmodal-shaped concentration profile. An additional factor could be that with increasing analysis time, the imperfections of the fluid-gel interface like a meniscus or other irregularities become less relevant relative to diffusion distance. The absolute values of the diffusion coefficients were slightly erratic until an analysis time of 60 min (see Figure 7A). Afterwards, they showed a moderately decreasing trend with only minor changes ( < 9%) happening after 120 min. As shown in Figures 7B,E, very similar trends could be observed for low-melt agarose with the absolute values of the diffusion coefficients being about 15% lower than in unmodified agarose.

To determine the statistical significance of differences between the diffusion coefficients of different agarose concentrations, a one-way analysis of variance (ANOVA) was performed for each time point and the resulting *p*-values plotted in Figure 7C. The ANOVA tested the null hypothesis that there was no significant difference between the mean values of *D* in hydrogels of different agarose concentrations. For *p* < 0.05, the null hypothesis can be rejected. This was the case for most data points of both modified and unmodified agarose beyond an analysis time of 180 min. The already described erratic nature of the first data points determined with low analysis times is also reflected in the corresponding *p*-values which fluctuate considerably until an analysis time of 105 min, where a continuous downward trend sets in resulting in overall minimum *p*-values at an analysis time of 240 min. As a consequence, an analysis time of 240 min was chosen for the final data evaluation.

The results show that despite the apparent moving boundary, the analysis time did not strongly affect the results of the diffusion coefficient estimation when the analysis time exceeded a certain minimum. However, the *p*-values at different analysis times imply that longer analysis times allow a more precise estimation of the diffusion coefficient which increases the statistical significance and hence relevance of the data.

The comparison of lysozyme diffusion coefficients in Figure 7F between the two types of agarose hydrogels and different agarose concentrations clearly shows the influence of the two analyzed parameters. For every concentration, the mean diffusion coefficient was higher in hydrogels from unmodified agarose than low-melt agarose, although the difference was only statistically significant for a concentration of 1.0% (w/w). There was also a clear trend of a decreasing *D* with increasing agarose concentration. For both unmodified and low-melt agarose, the difference of *D* between 0.5 and 1.5% (w/w) was statistically significant. These trends align well with data reported in literature. The diffusion coefficients of lysozyme and bovine serum albumin have been shown to decrease with increasing agarose concentration (0.5–3% (w/w)) (Liang et al., 2006). Higher concentrations of agarose lead to the formation of polymer networks with reduced mesh size and hence reduced diffusibility (Liang et al., 2006). The observation that the diffusion coefficient was higher in hydrogels of unmodified agarose than of low-melt agarose can be attributed to the structure of the respective polymer networks. Hydroxyethylation reduces the number of intrastrand hydrogen bonds (Zhang et al., 2018), leading to the formation of a tighter polymer mesh with smaller pores for the low-melt agarose, depending on the degree of substitution (Cook, 1982).

The absolute values for the estimated diffusion coefficients were between 0.80 ± 0.04 ×10^−10^ m^2^ s^−1^ for 1.5% low-melt agarose and 1.14 ± 0.02×10^−10^ m^2^ s^−1^ for 0.5% unmodified agarose. In good accordance with this, Ye *et al.* reported a value of 1.2 ± 0.01×10^−10^ m^2^ s^−1^ for the diffusion coefficient of lysozyme in an 0.5% agarose hydrogel at 21–24 °C (Ye et al., 2016). These results demonstrate the suitability of the presented method to provide diffusion coefficient estimates aligning well with previously reported literature values. The method is sensitive enough to allow the detection of statistically significant differences (*p* < 0.05) of the diffusion coefficient of lysozyme between samples of different agarose concentration (0.5–1.5% (w/w)). Also, the samples of unmodified agarose were found to be higher than the samples of low-melt agarose for every analyzed concentration (difference only statistically significant at 1.0% (w/w)), demonstrating the suitability to detect differences between slightly different material types.

The present study only investigated one analyte and a range of relatively similar hydrogels. The transferability of the method to other use cases and its applicability in a more general context should be investigated further. Hydrogel-analyte combinations with a very different rate of diffusion than observed here may require adaptations of the evaluation method or may not be suitable to be investigated with the presented setup at all. The use of hydrogels with smaller pores could reduce diffusion rates to an extent that the method is not sensitive enough for a valid diffusion coefficient estimation. The use of smaller analyte molecules with increased diffusion rate may allow shorter analysis times. In general, it is important to always consider possible interactions between analyte and hydrogel that could influence the observed diffusion rate.

### 3.3 Experimental Considerations

Previously reported methods for the determination of diffusion coefficients using UV area imaging sensors (Ye et al., 2011; Ye et al., 2012b; Jensen et al., 2014; Ye et al., 2016) employed large quartz cells requiring correspondingly large sample volumes. The high material consumption may be problematic in certain contexts like high-cost materials or during early-stage development when the amount of available material is very limited. The presented method requires only about 40 µl of hydrogel and 25 µl of analyte solution for one measurement which could be further reduced by optimizing the microfluidic chip design. The possibility of introducing variable channel heights for different analyte concentrations or investigating alternative chip materials, tailored to the requirements of the hydrogels and analytes to be investigated, adds to the appeal of employing microfluidic chips for this purpose.

However, several aspects of the applied experimental setup could be optimized. The error estimation (Section 2.9) demonstrated the importance of temperature regarding diffusional processes. The current setup did not allow any control over the temperature inside the microfluidic chip. Replacing the employed plastic casing for the microfluidic chip by a temperature-controlled metal casing (e. g., employing a Peltier element) could improve the reliability of the acquired data.

The employed microfluidic chip was designed to allow the observation of the hydrogel phase only. Shifting the imaging area would allow a direct investigation of the fluid-hydrogel interface and the observation of diffusional effects in the fluid phase. This could provide further insights into the effects of interface imperfections and the validity of boundary assumptions. In general, it is desirable to improve the quality and positioning of the hydrogel-fluid interface. The method of filling the channel with hydrogel, as employed in this study, produced non-straight and non-reproducible boundary layers due to the formation of a meniscus and slightly different fill levels. This could be resolved by using a chip consisting of two separate parts for the hydrogel and fluid phase. The part for the hydrogel could be overfilled and the hydrogel trimmed to form an appropriate interface, before the second part of the chip is attached and filled with the analyte solution. A chip-casing combination with less manufacturing tolerance could improve the alignment of the chip within the UV imaging system.

## 4 Conclusion

The method established in this study allows the estimation of diffusion coefficients for UV-absorbing analytes in transparent hydrogels. The diffusion of lysozyme through agarose hydrogels was observed using an ActiPix™ UV imaging system. To minimize material consumption, a microfluidic chip was employed, that reduced the required amount of hydrogel to 40 µl. Employing automated image and data processing in Matlab, the obtained raw absorbance data contained in video files were corrected and processed to generate lysozyme concentration profiles along the microfluidic channel. The concentration profiles were fitted with a solution of Fick’s second law to estimate diffusion coefficients. As a case study, the diffusion of lysozyme in hydrogels with different concentrations (0.5–1.5% (w/w)) of unmodified and low-melt agarose was analyzed. The obtained diffusion coefficients allowed the detection of significant differences between the different types and concentrations of agarose. Generally, the diffusion coefficients were higher in unmodified agarose and at lower concentrations of agarose. The estimated diffusion coefficient of lysozyme in 0.5% (w/w) agarose was in accordance with data reported by Ye *et al.* (Ye et al., 2016). Refinements of the experimental setup, especially regarding temperature control, could enhance the reliability of the obtained data further.

## Data Availability

The raw data supporting the conclusions of this article will be made available by the authors, without undue reservation.
